# An EEG-Based Identity Authentication System with Audiovisual Paradigm in IoT

**DOI:** 10.3390/s19071664

**Published:** 2019-04-08

**Authors:** Haiping Huang, Linkang Hu, Fu Xiao, Anming Du, Ning Ye, Fan He

**Affiliations:** 1 College of Computer, Nanjing University of Posts and Telecommunications, Nanjing 210023, China; 1216042916@njupt.edu.cn (L.H.); xiaof@njupt.edu.cn (F.X.); 1216042915@njupt.edu.cn (A.D.); yening@njupt.edu.cn (N.Y.); 1016041130@njupt.edu.cn (F.H.); 2 Jiangsu High Technology Research Key Laboratory for Wireless Sensor Networks, Nanjing 210023, China; 3 College of Computer Science and Technology, Nanjing University of Aeronautics and Astronautics, Nanjing 210016, China

**Keywords:** EEG, IoT, brainwaves, identity authentication, audiovisual paradigm, bagging ensemble learning

## Abstract

With the continuous increment of security risks and the limitations of traditional modes, it is necessary to design a universal and trustworthy identity authentication system for intelligent Internet of Things (IoT) applications such as an intelligent entrance guard. The characteristics of EEG (electroencephalography) have gained the confidence of researchers due to its uniqueness, stability, and universality. However, the limited usability of the experimental paradigm and the unsatisfactory classification accuracy have so far prevented the identity authentication system based on EEG to become commonplace in IoT scenarios. To address these problems, an audiovisual presentation paradigm is proposed to record the EEG signals of subjects. In the pre-processing stage, the reference electrode, ensemble averaging, and independent component analysis methods are used to remove artifacts. In the feature extraction stage, adaptive feature selection and bagging ensemble learning algorithms establish the optimal classification model. The experimental result shows that our proposal achieves the best classification accuracy when compared with other paradigms and typical EEG-based authentication methods, and the test evaluation on a login scenario is designed to further demonstrate that the proposed system is feasible, effective, and reliable.

## 1. Introduction

The rapid development of smart sensing devices, wireless communications, and mobile intelligent computing has prompted potential applications of Internet of Things (IoT) [1,2]; however, security issues have always been one of the concerns of IoT applications. Intelligent access control and identity authentication, which benefit from the close technological convergence of novel IoT terminals and machine learning, have emerged as indispensable components in some IoT applications such as the smart home, intelligent building, or safeguards for the purpose of security enhancement [3,4]. For example, the fully automatic private residence must verify the visitor’s authentication to ensure that all the unapproved entrances are prohibited. In order to achieve such an intelligent identity authentication, novel IoT devices and machine learning methods will be employed, which can facilitate intelligently sensing and processing data or signals collected from the environment. 

However, in traditional IoT applications, the prevalent methods for identity authentication include user passwords, device PINs, and RF cards. These authentication methods face the risks of being easily stolen, lost, or forgotten after passwords are updated. With the development of intelligent biometric authentication technologies, fingerprint recognition, gesture recognition, iris recognition, and facial feature recognition [5,6,7,8] have successively emerged in IoT application scenarios such as the smart home. Although these authentication methods utilize unique biometrics to improve security, they still suffer from the following challenges: (1) biological features can be replicated, which provides opportunities for attackers to deceive the current entrance guard system; (2) once body parts that carry these characteristics are damaged, recognition cannot be conducted in intelligent building; (3) it has some limitations for different types of visitors; for example, the disabled with no arms cannot use fingerprint recognition to open a door, and the blind cannot be identified by the iris.

In order to address the above problems, researchers have begun to focus on the novel IoT devices based on electroencephalography (EEG) signals [9], which can be used to authenticate in intelligent entrance systems for home or industry [10]. First, EEG signals are generated from the brain, and cannot be copied or stolen by current technologies. Second, since the individual’s brainwave is greatly affected by specific pressures or stimuli [11], an imposter‘s brainwave activities are usually ineffective. Furthermore, the brainwave has many special properties, such as high time resolution and high uniqueness [12]. Therefore, the authentication method based on EEG signal devices has significant advantages over the traditional authentication methods, and it can be applied in more reliable IoT authentication system based on machine learning technology [13].

Currently, the studies on EEG identity authentication are mainly divided into two categories: the methods focus on EEG signals generated by subjects who perform a simple task in a relaxed state, and those that focus on the EEG signals when subjects are stimulated by other external signals. The former not only brings signal interference, but also accelerates the subject’s fatigue, which causes the authentication accuracy to be much lower than the latter. Therefore, the latter is the concern of this paper. In addition, external stimulus signals may be single visual signals, such as images, text, and short video; and single auditory signals, such as shouts, whistles, and music pieces. These single stimuli signals have limitations for different types of visitors; for example, it is useless for visitors with visual or hearing impairments. Therefore, we aim at to design an EEG-based identity authentication system with an audiovisual paradigm including both visual and auditory signals.

The contributions of this paper can be summarized as follows:

1. Aiming at the deficiency of current EEG-based identity authentication methods, it proposes an audiovisual paradigm combining visual and auditory stimuli for an IoT application scenario. It can increase the stimulating intensity and then improve the recognition accuracy of visitors for an intelligent entrance system for home or industry.
2. Intelligent processing based on the collected EEG signals: in the pre-processing phase, aiming at the noise signals, reference electrodes, ensemble averaging, and independent component analysis (ICA) are adopted to remove artifacts; in the feature selection phase, an adaptive selection method is designed to void extracting invalid feature data, and the best-first search algorithm is performed to determine the optimal feature subset; in the classification recognition phase, the bagging ensemble learning method is executed to establish the best classification model based on naïve Bayes, logistic regression, and the back propagation (BP) neural network.
3. We evaluate the classification performance of three experimental paradigms through an analysis and comparison of the accuracy rate, precision rate, and false positive for each subject, and we verify the superiority of the audiovisual paradigm. Besides, our proposal is compared with other typical EEG-based systems based on the same dataset; the experimental results show that our proposal achieves better classification accuracy. Finally, in order to test the attack capacity of the impostor on the identity authentication, we simulated a login scenario of the intelligent entrance system, and the result demonstrates that our proposed EEG authentication system is feasible, secure, and reliable.

The remainder of this paper is organized as follows. Section 2 provides an overview of related work. Section 3 describes the test design and the experimental paradigm based on the intelligent entrance authentication system. The detailed system design and solution is present in Section 4, which includes the process of signal acquisition, pre-processing, feature selection, and classification recognition. The experimental evaluation and analysis are shown in Section 5. Finally, Section 6 concludes the whole paper.

## 2. Related Work

A series of experimental methods for detecting the characteristics of brainwaves under different conditions have appeared since the 1990s, which also have been used in IoT applications such as intelligent door monitoring and smart home. Their safety and reliability depend on the uniqueness of brainwave characteristics. As mentioned above, the current studies on EEG-based identity authentication can be mainly divided into two categories.

The first type is absorbed in the EEG signal’s generation, which requires subjects to carry out simple tasks in a relaxed state. Paranjape et al. [14] proposed recording EEG signals via a simple eyes-closed/eyes-open task in the relaxed state, and then realizing the biologic features recognition by establishing an autoregressive model. The experimental results achieved an average classification accuracy of 80%. Poulos et al. [15] also used an autoregressive model and the support vector quantization of brainwaves to achieve an accuracy of 84% when subjects performed a simple task in a relaxed state. Lan et al. [12] realized the authentication of 10 subjects for one access control system with the feature extraction and classification method CNN (convolutional neural network). Relying on the strong generalization and robustness of CNN, the experimental results showed that the recognition accuracy can reach up to 88% and 86% for eyes-closed (EC) and eyes-open (EO) tasks, respectively. Maiorana et al. [16] took a different approach by focusing on the analysis of a longitudinal database which is composed of the EEG traits in a resting state with both EC and EO; the results achieved an average classification accuracy of 85.6%.

The second EEG signal’s collection is based on the stimulus of external signals to subjects. Chen et al. [9] proposed an EEG login system based on rapid visual presentation. The tests let subjects quickly browse the randomly selected target images, and then classification was achieved through shrinkage discriminant analysis. The test results showed that the average classification accuracy could reach to 77.5 ± 5.9%. Gui et al. [17] proposed an experimental paradigm that required the subjects to silently read the correct words, wrong words, acronyms, and their own names. It extracted frequency features through wavelet packet decomposition and achieved classification via neural networks; it obtained an average accuracy of 90%. Palaniappan et al. [18] used visual evoked potentials to identify users with 90% average classification accuracy. They designed an experimental task where the images were presented in grayscale, and then analyzed the power variation in the gamma band.

According to previous studies, the recognition efficiency relying on the visual paradigm is better than that relying on the eyes-closed/eyes-open task, which is most likely because it will increase the signal noise when performing the eyes-closed/eyes-open task. However, these visual paradigms still have some defects in highly reliable IoT applications. For example, as to the experimental method of silent reading proposed by Gui et al., visual presentation cannot be realized if the authenticated user has dyslexia. In the rapid visual presentation experiment proposed by Chen et al., the identity authentication cannot be performed if the visitor is an achromate. In view of the above problems, an intelligent EEG identity authentication system based on audiovisual paradigm will be proposed, and these problems will be addressed well through intelligent data processing and machine learning algorithms.

## 3. Test Design and Experimental Paradigm

**Figure 1:**
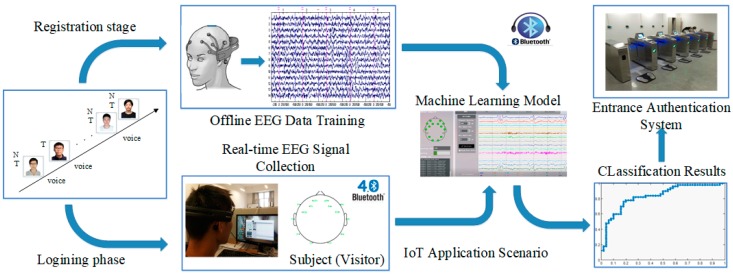
Electroencephalography (EEG)-based authentication system in Internet of Things (IoT) scenario.

As shown in Figure 1, the automatic entrance authentication system used in intelligent building is one of the most appropriate IoT application scenarios to verify the EEG-based design with an audiovisual paradigm. This scenario needs advanced brainwave signal devices that support Bluetooth 4.0 wireless communication [19], which is suitable for houses and buildings due to the characteristics of short-range and low-power consumption. The entrance authentication system can also support the Bluetooth signal receivers. In addition, the machine learning model will be integrated in the IoT scenario in order to achieve precise EEG feature matching and satisfactory authentication accuracy.

In this scenario, the individual authentication of visitors depends on the high uniqueness of brainwave signals. However, it is difficult to extract the distinctive features of brainwaves. In order to address this problem, we design the following experimental paradigm for an individual subject (visitor): induce the generation of the subject’s brainwave signals through watching their own face images and listening to the voice of their own name.

At the initialization phase, three target sources are selected by the subject, and 12 non-target sources are randomly selected by the entrance system from the material library. Each target source consists of the subject’s own face image and a voice calling their own name, while each non-target source involves another arbitrary subject’s face image and a voice calling the other arbitrary subject’s name.

According to studies of event-related potentials [20], the lower that the probability of a target stimulus appears, the greater the positive amplitude of the generated brainwave will be. Therefore, in order to enhance the stimulation intensity of the experiment, subjects are required to select their own face image for different periods as the target stimuli. After that, the subject needs to wait for the start sign of the experiment. There will be a short break at the end of each experimental paradigm, and each break consists of two phases. During the first phase, the subject is asked to enter the number of target sources that they count. This phase is merely aimed at improving the subject’s attention, but not using the input to do any further analysis. During the second phase, the subject can enjoy scenery pictures and have a 30-s rest with eyes closed at the end of each paradigm, in order to relieve the fatigue caused by the large number of stimuli from images and voice. Then, the subject can close their eyes for a moment until hearing the sound of “bi”. When the end sign is observed, the experiment is over. This experimental paradigm is implemented by the psychology experiment software E-Prime2.0, and data analysis is carried out via the EEGLAB toolbox.

## 4. Detailed System Design and Solution

**Figure 2:**
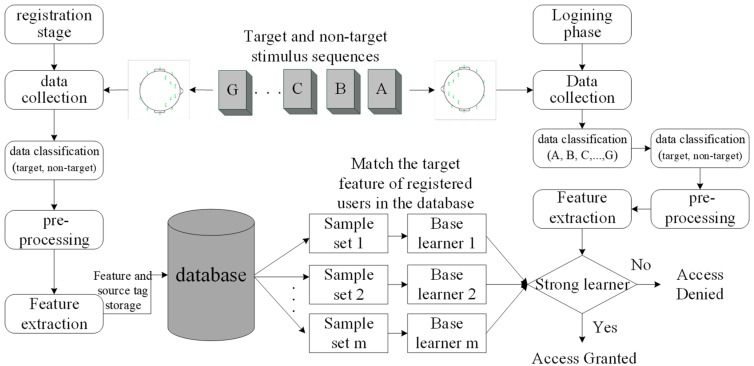
Three subsystems contained in the entrance authentication system.

Based on the experimental scenario of the audiovisual-inducing paradigm in Figure 1, the entrance authentication system may be composed of a registration subsystem, login subsystem, and authentication subsystem, whose functions are shown in Figure 2. 

In the registration phase, the first step is to acquire the signals. The subject selects the target source stimuli as a password, records the EEG signals that respond to the target source stimulus via the EEG device, and sends the EEG signal data to the terminal via a wireless Bluetooth module for further processing. The EEG signals are divided into target sources and non-target sources. Then, the target features and non-target features are extracted by pre-processing and feature extraction. In order to evaluate the performance of the system, some of these features are considered as training feature sets, and others are used as test feature sets. Finally, the target feature data set and source tag will be stored in the database.

In the login phase, EEG signals responding to multiple source stimuli will be recorded and then classified according to their respective source tags (A, B,..., G) and the machine learning model. Both processes of pre-processing and feature extraction are the same as those in the registration phase. After that, these signals with tags need to be grouped into a target source and non-target source.

In the authentication phase, according to the above grouping, the extracted target features will be matched with the registered user’s target features in the database. If it matches successfully, access is granted; otherwise, access is denied.

### 4.1. Signal Acquisition

The raw EEG signals are collected from 30 adult subjects (18 males and 12 females, their ages range from 21 to 45, the average age is 27.97, and the average deviation is 6.49), who will become the visitors to the intelligent building entrance system. None of them has a case history of brain injury or brain disease. When a subject is being authenticated, 29 other subjects can be randomly selected as impostors. As shown in the bottom left corner of Figure 1, the “EMOTIV EPOC+ EEG” head-worn device is employed as a novel IoT device, which has a total of 14 channels, namely: AF3, AF4, F3, F4, F7, F8, FC5, FC6, T7, T8, P7, P8, O1, and O2. The sampling frequency is 128 Hz, and the signals can generate 128 sample points per second per channel. Compared to the 64-channel EEG head-worn device, the equipment used in the experimental scenario is lighter, simple to wear, easier to use, and easier to popularize. Figure 1 also shows the software interface for brain signal processing.

### 4.2. Pre-Processing and Data Analysis

#### 4.2.1. EEG Signal Denoising

Due to the noise interference from raw EEG signals, such as EOG (electrooculography), power frequency, EMG (electromyography), and ECG (electrocardiography), ensemble average and independent component analysis (ICA) are adopted to pre-process the collected EEG signals in the experiment.

EOG is the most obvious blink noise affecting EEG signals. In the experiment, the AF3 and AF4 channels are sensitive to the blink of the subjects, so these two channels are used as the reference channel for blink detection. The signal whose maximum value exceeds the threshold value of 75 uV will be excluded, so as to reduce the disturbance caused by blink. However, this traditional method based on peak amplitude is unsuitable, because the change of the baseline voltage may cause the false and missed report. To address this issue, a peak-to-peak amplitude detection method is proposed, which measures the difference between the maximum and the minimum within the signal segment, and then compares the peak-to-peak voltage with the threshold voltage. This method is less likely to be distorted by the slow change of baseline voltage, which improves the sensitivity of the artifact correction process.

Secondly, for the interference caused by the power frequency and non-experimental stimulus, we adopt the ensemble averaging method to average the amplitudes [21]. The trend of the acquired EEG signal is a deterministic process, and that of the noise signal is an independent non-stationary process. It can be seen that after *n* times of stacking, the EEG-evoked potential value remains unchanged, while the noise signal is approximately zero. The calculation process is shown in Equation (1):

(1) $$\bar{x(t)}=\frac{1}{n}\sum_{i=1}^{n}x_{i}(t)=e(t)+\frac{1}{n}\sum_{i=1}^{n}N_{i}(t)=e(t)$$

(2) $$SNR=\sqrt{\sigma^{2}}/\sqrt{\sigma^{2}/n}=\sqrt{n}$$

In Equation (1), ***e***(*t*) is the denoised EEG signal set, *Ni*(*t*) is the noise signal, and *x*(*t*) is the ensemble average of the actual observed EEG signals x*_i_*(*t*) for *n* times. In Equation (2), *SNR* is the signal-to-noise ratio, *σ*^2^ is the variance of each observation signal, *σ*^2^/*n* is the ensemble average evoked response variance, and the signal-to-noise ratio is increased by $\sqrt{n}$ times.

Although the ensemble average is a simple signal processing method, it is efficient because the standard deviation of noise after the average is obviously reduced. In the experiment, the EEG signals is ensemble averaged eight times, and the *SNR* is increased by a factor of 2.8.

Both EMG and ECG signals are the most unique and intractable noise signals. Therefore, the independent component analysis (ICA) method is employed to deal with them [22]. In this process, independent signal sources are mixed by the mixing matrix to obtain actual observation records of EEG signals. However, in practice, we only know the observed signals, while the mixing matrix and independent signal sources are unknown. ICA can separate components of independent signal sources from observed signals under the premise that the mixed matrix and independent signal sources are unknown. Therefore, pure EEG signals, EMG signals, and ECG signals can be distinguished. ICA can be achieved via the EEGLAB toolbox.

**Figure 3:**
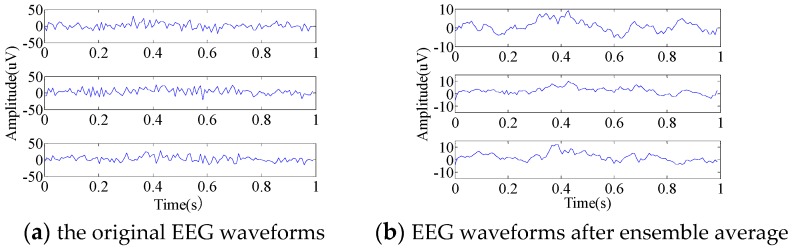
Comparison of the original brain wave and denoising brain wave.

Figure 3a gives three segments of the original EEG waveforms within one sampling period. We can see that the raw EEG signals contain a lot of noise, since the signals change rapidly. Figure 3b shows the curves after ensemble averaging. It can be seen that the averaged signals are much smoother than the raw data.

#### 4.2.2. Differential Analysis of EEG Data

**Figure 4:**
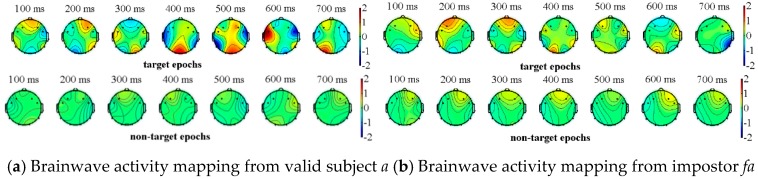
Brainwave activity mapping of target and non-target source stimulation from subject *a* and his corresponding impostor *fa*.

**Figure 5:**
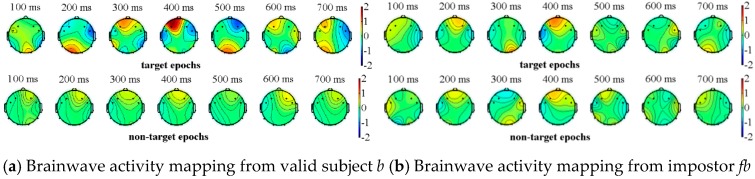
Brainwave activity mapping of target and non-target source stimulation from subject *b* and his corresponding impostor *fb*.

We completed the above experimental paradigm analysis on all of the subjects (visitors) where one impostor is randomly selected for each subject. Due to the space limitations, we only show the experimental results of two subjects and their corresponding impostors. The valid subjects *a*, *b* and their impostors *fa, fb* respectively perform the above-mentioned experiment, in which *a, b* select their own information as the target source, while the impostors *fa, fb* select the information of *a*, *b* as their target sources. The two types of event-related potentials can be obtained from Figure 4 and Figure 5, where the brainwave activity mappings of target and non-target source stimulation from valid subjects and their corresponding impostors can be depicted through the EEGLAB tool. In fact, by the observation of the brainwave activity mapping, the color change of the gradient pole (i.e., vertical bar in Figure 4) can be recorded. The amplitude of the brain wave in the current region, which is represented by the red color, shows a high-value state. On the contrary, the blue color represents the state in which amplitude is low. Among which the number at the top of the figures represents the current timestamp of the brainwave activity mapping.

As shown in Figure 4 and Figure 5, the brainwaves of valid subjects *a, b* have different responses to the target sources and the non-target sources. On the contrary, although the impostors are familiar with the target source information of the valid subjects, their brainwaves have no significant difference between the target sources and non-target sources. In view of this, event-related potentials can be used as the basis for feature extraction.

### 4.3. Pre-Processing and Data Analysis

For the classification of EEG signals, the concern is to extract highly reliable feature data from selected channels and time intervals. Due to neurophysiologic phenomena, the potentials caused by the self-sourced and non-self-sourced stimuli may appear significant differences in amplitude from a sampling point. However, due to the unpredictability of the cerebral cortex, there may exist differences for each channel and each time interval. In order to overcome this issue, the point biserial correlation coefficient method is adopted to determine the brainwave channels and time intervals for specific subjects [23].

#### 4.3.1. Calculation of the Point Biserial Correlation Coefficient

**Figure 6:**
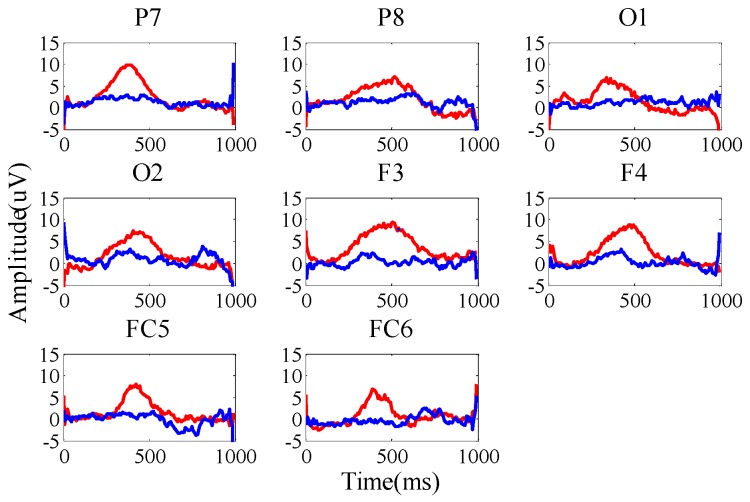
EEG-related potentials with target and non-target stimulation.

It can be seen from Figure 6 that there is a significant difference between the target feature and the non-target feature, and the point biserial correlation coefficient can accurately reflect the discrimination between the target sample and the non-target sample. In order to select the channels and time intervals with more obvious discrimination, this method was applied to the screening of brainwave channels and time intervals.

The point biserial correlation coefficient *r*(*t*) (at time *t*) can be calculated as shown in Equation (3):

(3) $$r(t)=\frac{\sqrt{n_{1}n_{2}}}{n_{1}+n_{2}}\cdot \frac{m_{1}(t)-m_{2}(t)}{S(t)}$$

where *T* is the length of each trial (*t*∈{1, ..., *T*}), and *n*_1_ and *n*_2_ represent the number of samples of the target trials and non-target trials, respectively. *m*_1_(*t*) and *m*_2_(*t*) represent the mean values of the potentials of all the target trials and those of all the non-target trials at time *t*, respectively. *S*(*t*) represents the standard deviation (the total sum of distance of each potential deviating from the mean) at time *t*.

According to Equation (3), the larger *r*(*t*) value is an important metric for selecting the channel and time interval. For each channel, the mean square value of *r*(*t*) in *T* time interval will be sorted in descending order. Then, the top eight channels will be selected. In our experiments, F3, F4, FC5, FC6, P7, P8, O1, and O2 are selected as the electrode channels. The event-related potential waveforms are shown in Figure 6.

The *r*(*t*) values of each sample point can be calculated according to Equation (3). The *r*(*t*) values of each current sample point together with its subsequent five sample points will be superposed and averaged. When the *r*(*t*) value of the current sample point is larger than the average value within *T* time interval and the duration interval is more than 195 ms (for a total of 25 sampling points), the feature data is extracted from this time interval. The corresponding time interval for each electrode channel is selected by our experiment, as shown in Table 1:

#### 4.3.2. Optimal Feature Subset Selection

In order to describe the characteristics of EEG signals, seven statistical features (f1–f7, see Table 2) are extracted from the training set and the test set for each subject. The reason for choosing these seven features in the experiment is that the mean value can reflect the central tendency of a sample data set; the median, although it cannot represent the whole sample, reflects the median level of the data; the standard deviation and the entropy can reflect the degree of dispersion of the sample; the maximum and the minimum are used to describe the distribution range of the sample, and the skewness can describe the degree of the sample data deviating from the mean. These features form together a set of feature vectors, but not all of the features are task-related. Therefore, a method combining feature search with performance evaluation is proposed to select the optimal feature subset.

The best-first method is used to search the feature subsets based on greedy hill-climbing augmented with a backtracking facility [24]; then, the correlation-based variable selection is used to assess the performance of the feature subset [25]. By comparing the EEG features of subjects, standard deviations and medians are selected as the optimal feature subset, which is denoted as Fs2. In order to verify the classification performance of this optimal feature subset, the other feature subset Fs1 will be compared, which includes all seven statistical features.

In the previous experiments, 54 target trials have been obtained, and eight channels have a total of 432 target trials. We can obtain 432 seven-dimensional target feature vectors and 432 two-dimensional target feature vectors from Fs1 and Fs2, respectively.

### 4.4. Classification Method

In order to obtain higher accuracy, the machine learning model adopts the bagging ensemble learning method, which needs to choose suitable base learners. An overview of some classic EEG schemes based on biometric systems is given in Table 3 in order to achieve the optimal choice of base learners.

In Table 3, the performance is represented by two metrics: CRR (the correct recognition rate) and HTER (the half total error rate). Considering the two metrics of these classifiers, the performances of the neural network, naïve Bayes, and logistic regression are better than the others. 

The main task of bagging is to reduce the variance of samples, so it is effective on the neural network learner that is susceptible to sample perturbation. At the same time, neural network is a nonlinear classifier and has always been a frequently used classification model in Brain Computer Interface (BCI) applications. In order to improve the generalization ability of the ensemble learning model, two linear classifiers—naïve Bayes and logistic regression—can present a better parallelization relationship. It is convincing that choosing these three classifiers in the experiments will achieve the satisfactory experimental results.

Firstly, we need to evaluate the performance of three classifiers. Then, we adjust the bagging algorithm according to the evaluation results. Besides, before training the classifier, the feature data set needs to be normalized. This is to avoid excessive influence on the result when a certain feature value is much larger than the other feature values.

#### 4.4.1. Naïve Bayes

The naïve Bayes classifier [26] is based on the naïve Bayes formula:

(4) $$P(c|d)=\frac{p(c)p(d|c)}{p(d)}$$

In Equation (4), *p*(*c*) is a priori probability, *p*(***d***|*c*) represents the conditional probability of sample *d* relative to class *c*, and *p*(***d***) is the evidence factor used for normalization.

In order to estimate the posterior probability *p*(*c*|***d***), the naïve Bayes classifier makes use of the “attribute conditional independence” assumption for the target and non-target samples. Since the attributes are independent of each other, Equation (4) can be rewritten as:

(5) $$P(c|d)=\frac{P(c)P(d|c)}{P(d)}=\frac{P(c)}{P(d)}\prod_{i=1}^{j}P(d_{i}|c)$$

(6) $$p(d_{i}|c)=\frac{1}{\sqrt{2\pi }\sigma_{c,i}}\exp(-\frac{{\left(d_{i}-u_{c,i}\right)}^{2}}{2{\sigma_{c,i}}^{2}})$$

In Equations (5) and (6), *j* represents the feature number of the feature set Fs1 or Fs2, *di* is the value of one of the samples ***d*** on the *i*-th feature, and *u_c,i_* and *σ*^2^*_c,i_* are respectively the mean and the variance of the *i*-th feature value of the two type samples.

#### 4.4.2. Logistic Regression

Logistic regression expects to establish a regression equation for the classification boundary line based on the existing feature data set Fs1 and Fs2, in order to find the best-fit parameter set [27]. It aims at classifying the target features and non-target features. The core functions of the classifier are shown as follows:

(7) $$logit(p)=ln(\frac{p}{1-p})=\beta_{0}+\beta_{1}w_{1}+\cdots +\beta_{n}w_{n}=\beta_{0}+\sum_{i=1}^{n}\beta_{i}w_{i}$$

(8) $$p(y=1|w_{1},w_{2},\ldots ,w_{n})=\frac{e^{\beta_{0}+\sum_{i=1}^{n}\beta_{i}w_{i}}}{1+e^{\beta_{0}+\sum_{i=1}^{n}\beta_{i}w_{i}}}$$

In Equations (7) and (8), *w*_1_, *w*_2_, ..., *w*_n_ are vectors of input features, and *y* is the corresponding class label that can be either zero or one, representing the target or non-target feature, respectively. *P* is the probability of occurrence of the event that *y* = 1. The relative event probability *P*(*y* = 0|*w*_1_, *w*_2_, ..., *w*_n_) can be calculated as 1 *– P*(*y* = 1|*w*_1_, *w*_2_, ..., *w*_n_). *β*_0_ is the intercept as a constant, *β*_1_, *β*_2_, ..., *β*_n_ are the regression coefficients related to the independent variables *w*_1_, *w*_2_, ..., *w*_n_ in a regression gradient rise optimization algorithm.

#### 4.4.3. BP Neural Network

The BP neural network model [28] consists of three layers: the input layer, the hidden layer, and the output layer, which are composed of neurons. The neurons of each layer are fully interconnected with those of the next layer, and there are no same-layer and cross-layer connections between neurons. The steps of the BP algorithm are described as follows:

1. Input samples are first provided to the neurons in the input layer, and then the signals are forwarded layer by layer until the neurons in the output layer get results.
2. According to the results, the error of the output layer will be calculated and back propagated to the hidden layer neurons.
3. Adjust the connection weights and thresholds between neurons based on the error in the hidden layer.
4. The loop iterates until it reaches the termination condition.

In the experiment, in the Fs1 feature set, there are seven neurons in the input layer, two neurons in the output layer, and four neurons in the hidden layer. In Fs2, there are two neurons in the input layer, two neurons in the output layer, and two neurons in the hidden layer. The training error is set as 0.01 (the selection of the training error is determined by comparison analysis between multiple measurements in the experiment).

#### 4.4.4. Bagging Ensemble Learning

Bagging is the most famous representative of the parallel-integrated learning method. The execution steps designed in our proposal are as follows:

1. Acquire the sample data set through the self-sampling method. The feature set extracted from this experiment contains 860 feature samples; 660 of them are randomly selected as training set D, and the remaining 200 samples are used as test sets. Every time, one sample is randomly selected from D and put it into the new sample set D’, and then put it back into D. The process is repeatedly executed 660 times to obtain a new sample set D’ with a total sample number of 660.
2. Repeat the above self-sampling process to obtain multiple sample sets, select one classifier for each sample set, and then perform training to obtain a base learner. Finally, all of the base learners will be integrated as a strong learner.
3. Bagging also uses a voting method for the classification task. The final prediction result is determined by the most counts of positive cases or negative cases provided by each base learner. If there are two classes receiving the same number of votes during the classification prediction, then one of them is selected randomly.

### 4.5. Performance Evaluation

The classifier performance can be evaluated using the precision rate *Tpr* (i.e., true positive rate), the false positive rate *Fpr*, and the accuracy rate *Acc*, which can be derived from Equations (9)–(11), respectively:

(9) $$Tpr=\frac{TP}{TP+FN}$$

(10) $$Fpr=\frac{FP}{FP+TN}$$

(11) $$Acc=\frac{TP+TN}{TP+FN+FP+TN}$$

*TP* stands for the number of target samples predicted to target samples, *FN* represents the number of target samples predicted to non-target samples, *FP* is the number of non-target samples predicted to target samples, and *TN* is the number of non-target samples predicted to non-target samples.

## 5. Experimental Test Results and Analysis

### 5.1. Selection of Optimal Classifier

Firstly, the three classifiers mentioned above are separately adopted to verify the performance of the two training feature sets (Fs1 and Fs2). Three classifiers use 10-fold cross-validation for performance evaluation so as to select high quality base learners for bagging ensemble learning. The respective classification results of the three classifiers for the feature set Fs1 and the feature set Fs2 are respectively shown in Table 4 and Table 5. We randomly selected seven subjects to show the classification results.

In Table 4 and Table 5, because of the difference in the state and concentration of each subject, there exist differences in the quality of the collected EEG signals, which might cause the classification accuracy of one subject to be higher than that of another. In addition, the classification performance of the selected optimal feature subset Fs2 (standard deviation and median) is significantly better than the set Fs1, which involved all of the extracted features through the respective verification of naïve Bayes, logistic regression (a.b. LR), and BP neural network classifiers.

At the same time, from Table 4 and Table 5, the classification performance of naïve Bayes is unsatisfactory compared with that of logistic regression and the BP neural network. In order to ensure the accuracy of the ensemble learner, the naïve Bayes model with poor classification performance was removed from the experiments. At the same time, considering that the number of base learners should not be too much and the voting method requires an odd number of base learners, the experimental choice combines three BP neural network base learners with two logistic regression ones into one strong classifier in the experiments. 

The performance of the classifier can be evaluated by the ROC curve and the AUC area, which are defined further in this paragraph. The ROC curve is called the “receiver operating characteristic curve”, which means that samples are sorted in ascending order according to the learner’s predicted results. All of the samples will be traversed and marked one by one in this order, and the current sample together with those marked samples will be regarded as positive samples to predict the remaining ones. The values of *Tpr* and *Fpr* are calculated and plotted on the vertical and horizontal coordinates, respectively. When comparing the learners’ performance, if the ROC curve of one learner is completely enveloped by the curve of another learner, the performance of the latter can be judged to be superior to the former. Besides, if the ROC curves of the two learners intersect, a more reasonable criterion is to compare the area under the ROC curve, that is, the AUC (area under the ROC curve).

**Figure 7:**
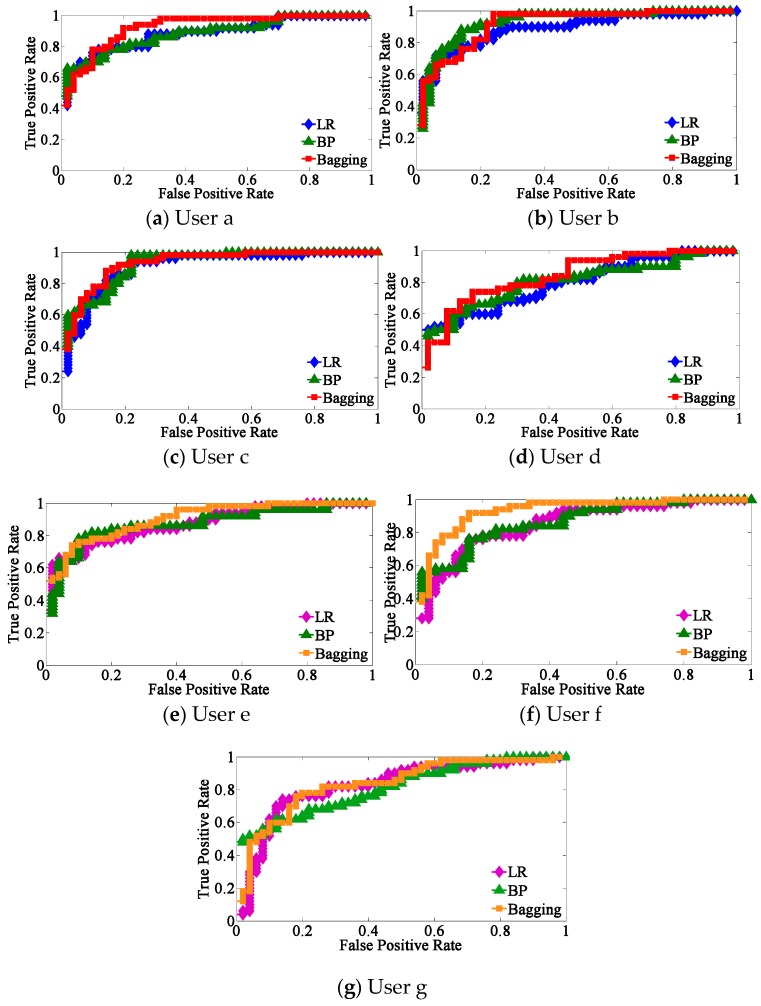
Comparisons of receiver operating characteristic (ROC) curves for the three classifiers from subjects (**a**–**g**).

**Figure 8:**
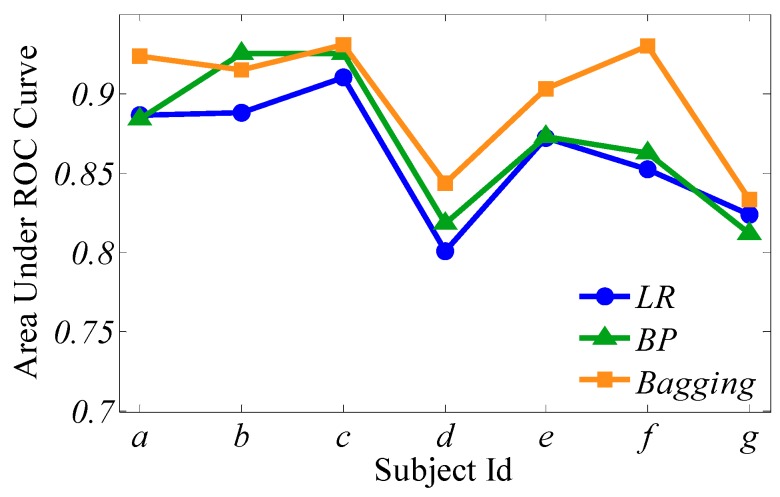
The area under the ROC curve (AUC) surrounded by the three learners of subjects *a*–*g*.

Figure 7 presents the comparisons of ROC curves for the three classifiers from the selected seven subjects a–g, and Figure 8 shows the AUC surrounded by the three learners from the same seven subjects.

It can be seen that the proposed bagging ensemble learning algorithm achieves a better classification effect whether from Figure 7 or Figure 8, followed by BP neural network and logistic regression.

### 5.2. Comparison of Experimental Paradigms

**Figure 9:**
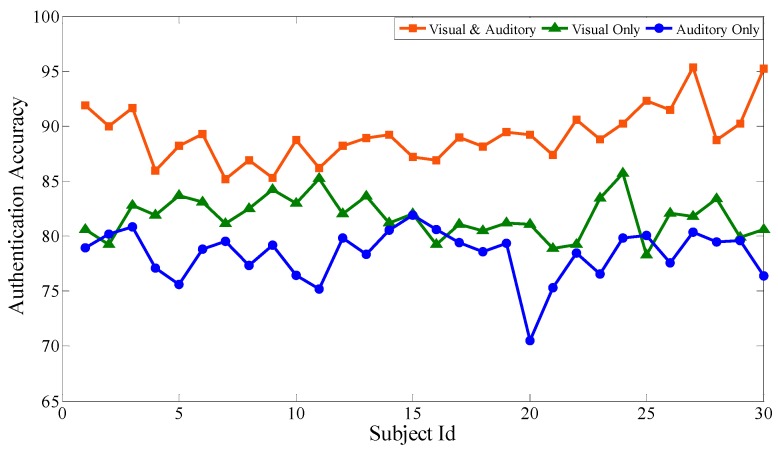
Comparison of average classification accuracy of the three paradigms.

**Figure 10:**
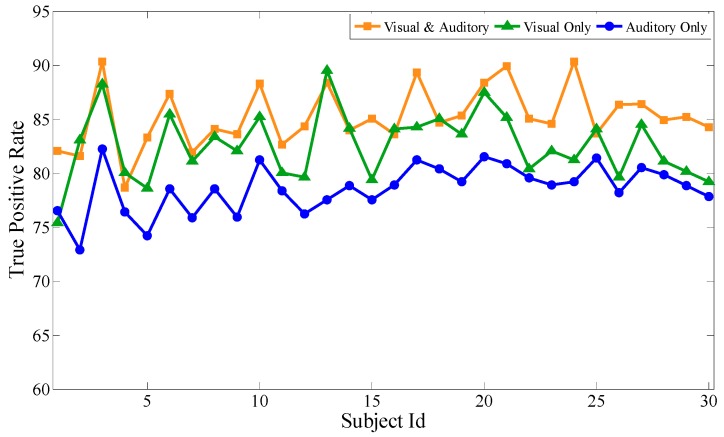
Comparison of the precision rate of the three paradigms.

**Figure 11:**
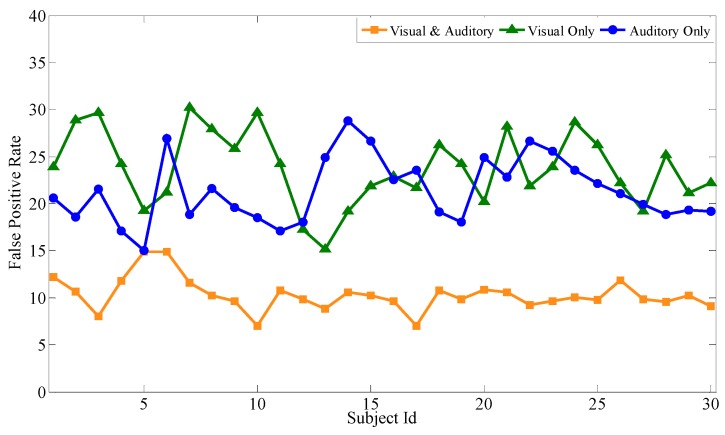
Comparison of the false positive rate of the three paradigms.

Based on the above experiments, the proposed feature selection method together with the bagging classification method forms the best combination, which will be used to evaluate the performance of the audiovisual paradigm, the single visual paradigm, and the single auditory paradigm, respectively. Similar to the audiovisual paradigm, the single visual paradigm and the single auditory paradigm will be conducted according to the experimental scenario in Section 3. Particularly, during the selection of stimuli source, only visual or auditory stimuli are selected. Except for this, the selection of optimal channels, optimal time intervals, and optimal feature subsets are all based on our proposal. Each subject performs the same experiment several times to obtain mathematical expectation. Aiming at 30 subjects, after 100 experiments, Figure 9, Figure 10 and Figure 11 respectively show the average classification accuracy rate, precision rate (true positive rate), and false positive rate of the three paradigms.

From the comparison of the accuracy rates of the three experimental paradigms, the audiovisual paradigm achieves the best classification effect. Based on the analysis of the true positive rate and the false positive rate, although the true positive rate of the audiovisual paradigm has no obvious advantage over the other two paradigms, its false positive rate is significantly lower than the other two. For the entrance identity authentication system for a smart building or house, the false positive rate is an important metric to prevent identity authentication attacks from impostors. It can be seen that the combination of visual and auditory paradigms can actually increase the stimulation intensity compared with the single visual paradigm and the single auditory paradigm, ensuring that the extracted feature data is more reliable while obtaining better authentication effectiveness. At the same time, single visual or single auditory paradigms can also achieve desirable identity authentication for the visitors with only visual or auditory impairments.

### 5.3. Comparison of Experimental Methods

The first compared method was proposed by Gui et al. [17] based on wavelet packet decomposition and artificial neural network. In this experiment, the EEG signals were first ensemble averaged. After that, a 60-Hz low-pass filter was adopted to remove the noise out of the major range of the EEG signals. Then, the four-level wavelet packet decomposition is performed on each data set. After that, EEG signals can be separated into five major frequency bands signals: 0–4 Hz, 4–8 Hz, 8–15 Hz, 15–30 Hz, and 30–60 Hz. Then, the mean, the standard deviation, and the entropy of these frequency bands signals are calculated to form the feature data set. Finally, the feature data set is input into the artificial neural network for classification and authentication.

The second compared method was proposed by Chen et al. [9] based on the non-overlapping time window and shrinkage discriminant analysis. For the Event-related potential (ERP) analysis, the EEG data was low-pass filtered by a Chebyshev digital filter with a passband of 40 Hz and a stopband at 49 Hz. Features were calculated from 16 electrode channels by averaging voltages in nine non-overlapping time windows with a width of 100 ms, starting from 100 ms to 1000 ms with respect to stimulus onset. This resulted in 16 × 9 = 144 dimensional feature vectors. Then, the feature vectors are input into the shrinkage discriminant analysis classifier for classification recognition.

The third compared method was proposed by Wen et al. [29] based on boosting for transfer learning. They proposed a novel framework TrAdaBoost for transferring knowledge from one distribution to another by boosting a basic learner. They used support vector machines as the basic learners in TrAdaBoost. The basic idea is to select the most effective diff-distribution instances as additional training data for predicting the labels of the same distribution.

The first two methods above only pay attention to feature extraction while ignoring the further assessment and selection for the given features. Besides, only one classifier is employed by both.

Although the third one is a kind of ensemble learning method, boosting takes advantage of the relevance of the base learner, and the next learner utilizes the previous result, so it can only be sequential but not parallel, and finally it can be achieved by updating the weight of the training sample.

In order to verify the validity of our proposal based on bagging ensemble learning, the experiment selected seven subjects to collect their EEG data based on the audiovisual paradigm; these data will be regarded as the experimental data set for the fair comparisons. In the experiment, the data set must be pre-processed according to our proposal and the above three methods, respectively. After that, feature extraction and classification recognition are completed separately. Finally, 100 experiments for each subject are performed, and the average classification accuracy of the four methods is shown in Table 6.

As can be clearly seen from Table 6, the accuracy of our proposal for the seven selected subjects is higher than that of the three other methods. That means the adaptive feature selection and bagging ensemble learning method designed in this paper have more satisfactory performance compared with the traditional feature extraction and classification recognition methods.

### 5.4. Simulating Login in IoT scenario

In order to test the attack capacity of the impostor on the entrance authentication system of an intelligent building or residence, we deploy a well-trained ensemble learning model to a test server, and we can acquire and send the subject’s EEG signals through Bluetooth and use the test server as an intelligent gateway to implement entrance access control. There are 10, 30, and 100 subjects to perform simulated login; each of them will try to log into the authentication system and randomly select another subject as an impostor. As mentioned above, both the valid subject’s and the impostor’s target features are extracted under the valid subject’s self-source stimulation. Then, the target features of the valid subject and that of the impostor in the login stage will be respectively matched with that of the valid subject in the registration phase. 

**Figure 12:**
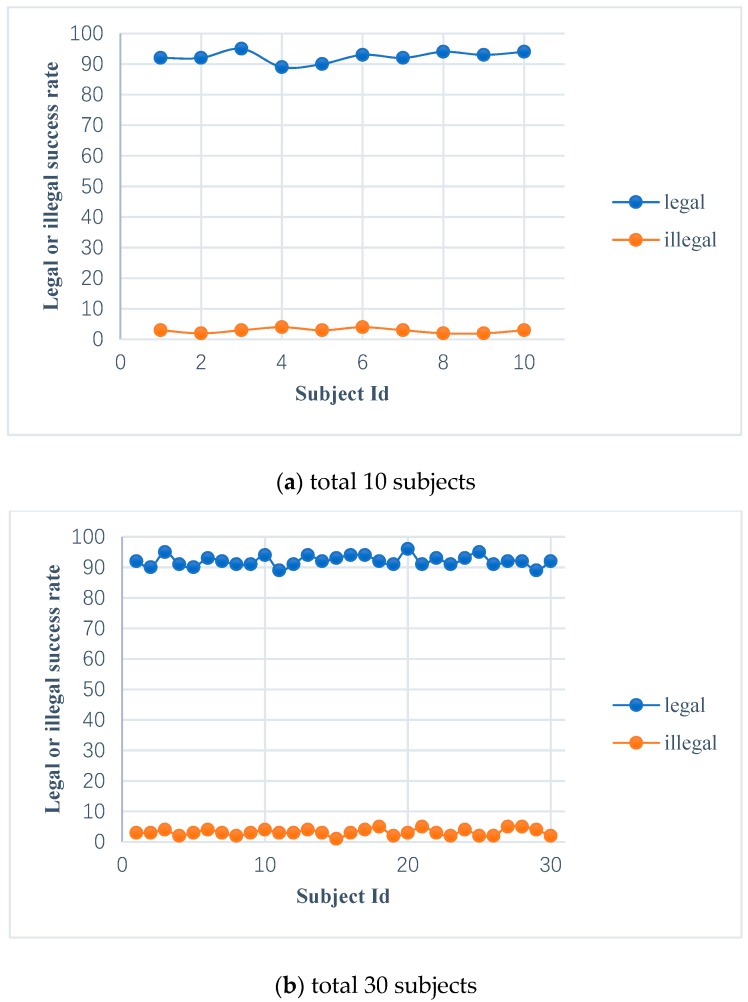

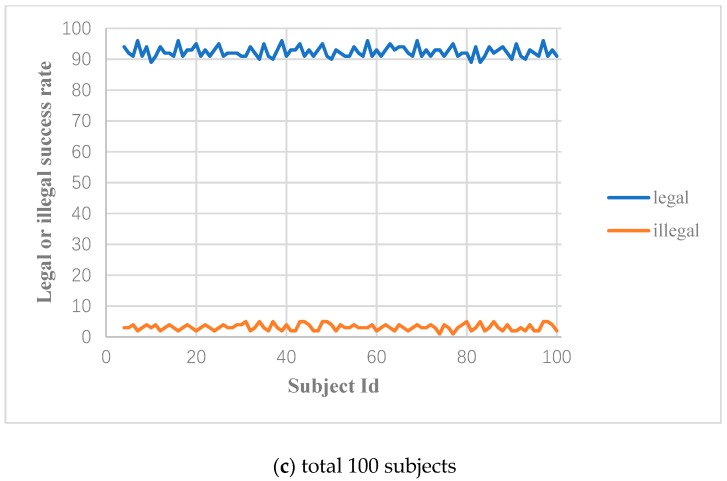
The legal and illegal success rate of the valid subjects and impostors.

In the experiment, mathematics expectations can be achieved by carrying out 10 experiments for each subject. Figure 12 shows the legal success rate (the probability of the valid subject passing the authentication) and the illegal success rate (the probability of the impostor passing the authentication) of the subjects. The legal success rate of 10, 30, and 100 subjects are 92.4%, 92.13%, and 92.42%, respectively, and the illegal success rate of 10, 30, and 100 subjects are 2.9%, 3.2%, 3.17%, respectively. From the perspective of security, the higher legal success rate and the lower illegal success rate are expected by the entrance authentication system, which verifies the reliability and effectiveness of the authentication system. 

### 5.5. Discussion

Through the above simulation experiments and analysis, our proposal based on the “EMOTIV EPOC+” EEG device still has a certain failure rate; we summarize the main reasons as below.

In our experiments, the subjects perform the visual and auditory paradigm in a open environment due to the constrains of experimental conditions, so there are many uncertainties in the test process: (1) the mental state of the subject may be bad; (2) the subject cannot correctly follow the authentication instructions of the experimental paradigm; (3) improperly wearing the EEG device can cause inaccuracies in the data acquisition; or (4) the surrounding noises may interfere with the subject. Furthermore, we find that the 64-electrode EEG devices may achieve the higher precision but they are expensive; for example, the price of the NeuroScan device is between $13,000–15,000. In contrast, the cost of our proposed EEG device is not very high; therefore, the accuracy of the collected data has a certain limitation.

Based on the above analysis, some difficulties exist in popularizing brainwave-based authentication methods. We suggest the following solutions to achieve more satisfactory experimental results.

First, the subject needs to be fully acquainted with the workflow of the audiovisual paradigm and strictly perform the instructions.

Second, the subject needs to be in a confined space and comfortable state to avoid the surrounding interference.

Finally, promoting the accuracy of data collection by continuously improving the experimental paradigm under the premise of controllable cost. Optionally, we may also adopt more high-precision data acquisition devices if not counting the cost.

## 6. Conclusions and Future Work

The development of IoT device and artificial intelligence (AI) technology enables the traditional scenario to emerge for promising individual/industrial applications. This paper focuses on the security of an intelligent entrance guard for a smart home or building, and proposes an EEG-based identity authentication system that combines both visual and auditory presentations. Among which, the stimulus source including self and non-ego face images and voice can stimulate the subject to produce unique brainwave activity.

Based on the collected EEG signals, various well-designed methods were selected to achieve artifact removal and construction of the optimal feature subset. Then, base learners that were selected from naïve Bayes, logistic regression, and BP neural network were integrated to a strong learner using the bagging ensemble algorithm for the classification performance evaluation of feature subsets. Furthermore, lots of experimental analyses were carried out to verify the feasibility, effectiveness, and reliability of our proposed identity authentication system in order to satisfy the requirements of IoT applications. 

The proposed EEG-based identity authentication system has characteristics that cannot be forged or replaced, which is even appropriate for the special visitor whose brain activity is normal but has poor eyesight or hearing such as a blind person or deaf person. However, how to achieve higher classification accuracy and more reliable authentication efficiency has become the concern of our future work. We will combine our proposal with other biometric technologies to form a more effective and secure authentication system.

## Figures and Tables

**Table 1:** Selected channels and time intervals.

Channel	Time Interval (ms)	Channel	Time Interval (ms)
P7	195–609	F3	273–648
P8	242–625	F4	289–703
O1	258–515	FC5	328–585
O2	242–562	FC6	281–539

**Table 2:** Statistical features.

Feature	Feature Description	Feature	Feature Description
f1	Standard deviation	f5	Minimum
f2	Skewness	f6	Mean
f3	Entropy	f7	Median
f4	Maximum		

**Table 3:** Overview of some classic EEG schemes based on biometric systems. CRR: correct recognition rate, HTER: half total error rate.

Paper	Protocol	The Number of Subjects	Channels	Features	Classifier	Performance
Gui et al. [17]	Read silently the words	32	6 (Fpz, Cz, Pz, O1, O2, Oz)	wavelet packet decomposition	Neural Network	CRR = 90%
Yeom et al. [23]	Visual evoked potentials	10	18	dynamic feature	Support Vector Machine	CRR = 86.1%
He et al. [26]	Motion tasks	4	19	Multi-variate autoregressive (mAR) features	Naïve Bayes	HTER = 8.1%
Subasi et al. [27]	24-h EEG recorded	5	4 (F7-C3, F8-C4, T5-O1, T6-O2)	wavelet transform analysis	Neural Network	CRR = 92%
					Logistic Regression	CRR = 89%
Marcel and Millan [11]	Word generation	9	8 centro-parietal	Gaussian mixture model	Maximum A Posteriori (MAP) model adaptation	HTER = 12.1%

**Table 4:** The classification results based on the seven-feature set (Fs1). ACC: accuracy rate, TPR: true positive rate, FPR: false positive rate.

Subject	Naïve Bayes			Logistic Regression			BP Neural Networks		
	*Acc*(%)	*Tpr*(%)	*Fpr*(%)	*Acc*(%)	*Tpr*(%)	*Fpr*(%)	*Acc*(%)	*Tpr*(%)	*Fpr*(%)
*a*	78.02	75.11	19.07	78.49	77.67	20.7	79.07	74.42	16.28
*b*	78.84	77.21	19.53	82.56	83.72	18.6	84.88	90.70	20.93
*c*	81.40	76.74	13.95	85.12	83.72	11.16	86.51	84.18	13.49
*d*	74.42	76.74	27.9	77.90	75.35	19.53	77.91	81.40	25.58
*e*	77.79	75.12	19.53	80.23	69.77	9.30	81.98	78.14	14.19
*f*	78.14	76.05	19.76	82.56	81.40	13.95	83.72	81.40	16.28
*g*	77.09	73.02	18.84	84.30	81.63	12.09	84.76	83.49	13.89
*Average*	77.96	75.71	19.80	81.59	79.04	15.05	82.69	81.96	17.38

**Table 5:** The classification results based on the two-feature set (Fs2). BP: back propagation.

Subject	Naïve Bayes			Logistic Regression			BP Neural Networks		
	*Acc*(%)	*Tpr*(%)	*Fpr(%)*	*Acc*(%)	*Tpr*(%)	*Fpr*(%)	*Acc*(%)	*Tpr*(%)	*Fpr*(%)
*a*	85.00	92.79	22.79	86.40	85.11	12.32	87.91	86.05	10.23
*b*	84.42	92.56	23.72	86.40	84.65	11.86	86.98	85.58	11.63
*c*	87.67	90.70	15.35	89.30	87.91	9.30	89.65	92.33	13.02
*d*	75.93	71.86	20.00	78.72	77.21	19.77	80.93	77.67	15.81
*e*	82.56	89.77	24.65	85.23	83.26	12.33	85.47	82.33	11.86
*f*	83.95	91.16	23.26	87.44	85.81	10.93	88.26	88.37	11.86
*g*	84.07	91.16	23.02	86.60	88.14	10.93	88.16	80.93	8.60
*Average*	83.37	88.57	21.83	85.73	84.58	12.49	86.77	84.75	11.86

**Table 6:** Performance comparisons with the existing methods.

Authors	Methods	Classification Accuracy Rate (%)							
		a	b	c	d	e	f	g	Average
**Our proposal**	**Bagging with adaptive feature selection**	**91.91**	**91.98**	**93.65**	**89.93**	**90.23**	**92.26**	**90.16**	**91.45**
Gui et al. [17]	Artificial Neural Networks (ANNs) with Wavelet packet decomposition (WPD)	87.31	85.74	86.10	88.81	84.12	85.02	86.95	86.30
Chen et al. [9]	Shrinkage Linear Discriminant Analysis (LDA)	85.23	85.14	84.45	87.14	83.90	86.95	83.64	85.21
Wen et al. [29]	Boosting for transfer learning	88.61	89.32	91.28	89.46	85.81	88.18	87.35	90.56

## Data Availability

All data used in this manuscript are collected from subjects by the electrical equipment of “Emotiv Epoc+”. The data sets generated and analyzed during the current study are available from the corresponding author on reasonable request.
